# US State Policies and Mental Health Symptoms Among Sexual and Gender Minority Adults

**DOI:** 10.1001/jamanetworkopen.2025.12189

**Published:** 2025-05-23

**Authors:** Briana S. Last, Nguyen K. Tran, Micah E. Lubensky, Juno Obedin-Maliver, Mitchell R. Lunn, Annesa Flentje

**Affiliations:** 1 Department of Psychology, Stony Brook University, Stony Brook, New York; 2 The PRIDE Study/PRIDEnet, Stanford University School of Medicine, Palo Alto, California; 3 Department of Obstetrics & Gynecology, Stanford University School of Medicine, Stanford, California; 4 Stanford Prevention Research Center, Department of Medicine, Stanford University School of Medicine, Stanford, California; 5 Department of Epidemiology and Population Health, Stanford University School of Medicine, Stanford, California; 6 Division of Nephrology, Department of Medicine, Stanford University School of Medicine, Stanford, California; 7 Alliance Health Project, Department of Psychiatry and Behavioral Sciences, School of Medicine, University of California, San Francisco; 8 Department of Community Health Systems, School of Nursing, University of California, San Francisco

## Abstract

**Importance:**

A recent increase in state policies targeting gender minority (GM; transgender and gender-diverse) people may affect the mental health of sexual and gender minority (SGM; nonheterosexual and/or GM) people and GM people specifically.

**Objective:**

To estimate changes in mental health symptoms associated with enactment of anti-GM state policies among SGM people and GM people specifically.

**Design, Setting, and Participants:**

This repeated cross-sectional study used a staggered difference-in-differences analysis to examine the associations between anti-GM policies in the US and mean changes in mental health symptoms among 8733 SGM adults who completed annual questionnaires between April 1, 2020, and June 1, 2023, for The Population Research in Identity and Disparities for Equality (PRIDE) Study, a national, prospective, continuously enrolling online cohort study of SGM adults.

**Exposures:**

Living in a state with 1 or more of the following enacted policies: (1) bathroom restrictions for GM people, (2) sports bans for GM young people participating in school sports, and (3) bans on gender-affirming care for young people.

**Main Outcomes and Measures:**

Mean levels of anxiety (measured using the 7-item Generalized Anxiety Disorder scale [GAD-7]; total score range, 0-21), depression (measured using the 9-item Patient Health Questionnaire scale [PHQ-9]; total score range, 0-27), and posttraumatic stress disorder (PTSD) symptoms (measured using the 6-item PTSD Checklist scale [PCL-6]; total score range, 6-30). For all 3 scales, higher scores indicate more severe symptoms.

**Results:**

Among all 8733 SGM participants in the sample (median age, 32.5 years [IQR, 26.0-45.0 years]; 2024 cisgender men [23.2%], 2355 cisgender women [27.0%], 2198 gender-diverse adults assigned female at birth [25.2%], 321 gender-diverse adults assigned male at birth [3.7%], 1294 transgender men [14.8%], and 541 transgender women [6.2%]), anti-GM policy enactment was associated with significant increases in anxiety (GAD-7 score, 0.8 points [95% CI, 0.2-1.4 points]) and PTSD (PCL-6 score, 0.8 points [95% CI, 0.1-1.4 points]) symptoms in states that enacted anti-GM policies compared with states that did not but was not associated with significant increases in depression symptoms (PHQ-9 score, 0.6 points [95% CI, −0.1 to 1.4 points]). In the GM subsample (n = 4354), nonsignificant changes in anxiety (GAD-7 score, 0.6 points [95% CI, −0.2 to 1.4 points]), depression (PHQ-9 score, 0.1 points [95% CI, −0.9 to 1.1 points]), and PTSD (PCL-6 score, 0.7 points [95% CI, −0.2 to 1.6 points]) symptoms were observed after policy enactment in states that enacted anti-GM policies compared with states that did not. Gender minority adults had high mental health symptoms across the study period.

**Conclusions and Relevance:**

In this study of 8733 SGM adults using difference-in-differences analysis, anti-GM policies were associated with worse mental health symptoms among SGM adults but no changes in mental health symptoms among GM adults. As these policies proliferate, it is important to consider how they may affect mental health.

## Key Points

**Question:**

Are policies targeting gender minority (GM; transgender and gender-diverse) people associated with more mental health symptoms among sexual and gender minority (SGM; nonheterosexual and/or GM) people and GM people specifically?

**Findings:**

In this repeated cross-sectional study including 4354 GM adults within the total sample of 8733 SGM adults in a national cohort study, The PRIDE Study (2020-2023), anti-GM policy enactment was associated with significant increases in anxiety and posttraumatic stress symptoms and nonsignificant increases in depression symptoms among SGM adults and smaller nonsignificant increases in all symptoms among GM adults. Gender minority adults had high mental health symptoms overall.

**Meaning:**

This study suggests that anti-GM policies are associated with worse mental health among SGM people.

## Teaser

This cohort study uses a difference-in-differences analysis to estimate changes in mental health symptoms associated with enactment of anti–gender minority state policies among sexual and gender minority people and gender minority people specifically.

## Introduction

In the past several years, there has been an increase in US state policies restricting the health care access and civil rights of gender minority (GM; transgender or gender-diverse) people.^1,2,3^ In the 2024 legislative session, more than 500 anti-GM bills were introduced across 40 states, making 2024 the year with the most anti-GM legislation in US history.^4^ These policies have, for example, prohibited GM people from using bathrooms congruent with their gender identity, banned gender-affirming care (GAC) for young people, and banned GM young people from participating in K-12 and college sports.^2,5,6,7^ The increase in these policies has been sudden: before 2021, no state had passed a law banning GAC for GM young people; by the time of writing, 27 states now restrict these practices.^5^ Beyond state policies, federal policies restricting GM people’s rights are rapidly proliferating.^8^

Research suggests that recent anti-GM policies are associated with increased mental health symptoms among those directly affected by these policies (ie, GM young people).^3,9,10,11,12,13,14^ Anti-GM policy enactment between 2018 to 2022 was associated with 7% to 72% increases in past-year suicide attempts among GM people aged 13 to 24 years.^10^ Less research has examined how these policies may be associated with the mental health of sexual and gender minority (SGM) people more broadly—that is, nonheterosexual and/or GM people (such as lesbian, gay, bisexual, and transgender people)—who may be indirectly affected by or fear these policies’ repercussions. Cross-sectional studies suggest that anti-GM policies are associated with worse mental health among SGM people not directly targeted by the policies. A 2023 survey of 14 000 SGM adults found that 79% reported feeling less safe due to GAC bans, which have largely targeted youths.^15^ Similarly, a cross-sectional survey of 797 GM adults found that those worried about having their rights taken away had greater odds of anxiety and depression.^11^ This emerging evidence suggests a need to comprehensively understand how anti-GM policies are associated with SGM adults’ mental health.

To evaluate the associations between these anti-GM policies and the mental health of SGM and GM adults, we examined data from the largest dynamic (ie, continuosly enrolling), community-engaged, prospective cohort study of SGM adults from all 50 US states and its territories, The Population Research in Identity and Disparities for Equality (PRIDE) Study. Leveraging this large online study, we conducted a difference-in-differences analysis capturing staggered enactment of anti-GM policies to enhance our precision in estimating the associations of these policies with SGM mental health symptoms including anxiety, depression, and posttraumatic stress disorder (PTSD) symptoms. We analyzed these policy and mental health associations among SGM adults and GM adults, expecting that these policies would be associated with worse mental health symptoms for all SGM people but would be more pronounced among GM adults.

## Methods

The PRIDE Study is approved by the WIRB-Copernicus Group, Stanford University, and University of California, San Francisco institutional review boards. All participants provided electronic informed consent. This repeated cross-sectional study followed the Strengthening the Reporting of Observational Studies in Epidemiology (STROBE) reporting guideline.^16^

### Study Design and Participants

Participants in The PRIDE Study are invited to complete annual questionnaires examining their social experiences and health.^17^ Participants must reside in the US or its territories, be 18 years or older, identify as SGM, and read and understand English. Details about community engagement, recruitment, and data collection are described elsewhere.^18,19^

We included 4 waves of repeated cross-sectional annual data collected from 11 462 participants between July 2019 and June 2023 (2019 through 2022 annual questionnaires). Annual questionnaires typically start in June or July and end in May or June of the next year. We included all available data from The PRIDE Study participants who completed their annual questionnaires during the study period with 2 exceptions: (1) we restricted the sample to those who started their questionnaire on or after April 1, 2020, to avoid measuring changes concomitant with the onset of the COVID-19 pandemic, and (2) we excluded participants with missing data for any of the 3 mental health symptom measures of interest (described below), state of residence, or gender identity. Excluded participants had a higher percentage of missing income data but were otherwise similar to the analytic sample (eTable 1 in Supplement 1).

### Exposure

We operationalized exposure to anti-GM policies as residing in a state with 1 or more of the following anti-GM policies enacted in a specific survey year: (1) prohibitions on GM people using bathrooms congruent with their gender identity, (2) bans on GAC for young people, and (3) bans on GM young people participating in sports. We documented the earliest instance of these anti-GM state policies using publicly available policy databases from the Movement Advancement Project, the American Civil Liberties Union,^4,5,6,7^ and state websites.

Many states enacted several anti-GM policies during the same survey year. For states with multiple sequential policy enactment, we selected the first enactment date of any anti-GM policy because we were interested in estimating the mental health associations of a state’s sociopolitical climate rather than the associations of a specific policy. For our lagged analyses (see below), this resulted in 2 exposure groups based on the survey year in which the first anti-GM policy was enacted: enactment during the 2020 and 2021 annual questionnaire administration time periods. We used survey month and year to classify exposure period in states with enacted policies. For states with anti-GM policy enactment in 2020, their first exposure period was 2021; for states with anti-GM policy enactment in 2021, their first exposure period was 2022. States that enacted policies in 2022 were not considered exposed in the study period, as subsequent survey data on mental health symptoms were unavailable at the time of analysis. A list of states in each exposure and comparison group is shown in eTable 2 in Supplement 1.

### Outcomes

The primary outcomes were mean levels of anxiety, depression, and PTSD symptoms measured in each of the 2019, 2020, 2021, and 2022 annual questionnaires. Participants self-reported anxiety and depression symptoms “over the last 2 weeks” on the 7-item Generalized Anxiety Disorder scale (GAD-7; total score range, 0-21)^20^ and 9-item Patient Health Questionnaire (PHQ-9; total score range, 0-27),^21^ respectively. They reported PTSD symptoms “in the past month” on the 6-item PTSD Checklist (PCL-6; total score range, 6-30).^22^ For all 3 scales, higher scores indicate more severe symptoms.^17^

### Sociodemographic and State-Level Characteristics

Sociodemographic and state-level characteristics included age, gender identity, ethnic and racial identity, sexual orientation, annual household income, residence in a Medicaid expansion state as of 2020 per Kaiser Family Foundation data, and Bureau of Labor Statistics 2020 state-level unemployment rates.^23,24^ Because participants could have completed multiple questionnaires during the study period, we used participants’ first completed questionnaire to characterize the sample descriptively. Participants self-reported their identities; all annual questionnaire questions, including the demographic questions, are available online.^17^ Participants could select multiple options for their ethnic and racial identity, sexual orientation, and gender identity, with an option to select “another” and write in a response. Participants’ self-reported ethnic and racial identities were collected for the current study to describe the sample’s demographic characteristics. Gender subgroups were based on a 2-step procedure that cross-stratified gender identity and sex assigned at birth to create 6 mutually exclusive categories (cisgender man, cisgender woman, gender-diverse adult assigned female at birth, gender-diverse adult assigned male at birth, transgender man, and transgender woman).^25^ Participants who selected noncisgender identities were categorized as GM.

### Statistical Analysis

To describe the overall sample and comparison groups, we summarized continuous variables with mean values, median values, and their respective dispersion measures and categorical variables with frequencies and percentages. A difference-in-differences approach with staggered treatment enactment, developed by Callaway and Sant’Anna,^26^ was performed to assess whether participants’ mean levels of anxiety, depression, and PTSD symptoms were higher after states enacted anti-GM policies compared with participants’ mean symptom levels in a comparison group (while accounting for differences in the timing of policy enactment across multiple survey years).^27^ The comparison group included states not exposed to anti-GM policies during the study period.

Difference-in-differences coefficients were estimated using multivariable linear regression with covariate adjustments and inverse probability weighting to account for potential confounding (ie, a doubly robust approach).^28^ Covariate adjustments included age, annual household income, Medicaid expansion state status, and state-level unemployment rates. This approach estimates the weighted mean of all group-time estimates with weights proportional to the group size (ie, the number of observations in each exposed group). The 95% CIs were estimated using a multiplier bootstrap procedure that is clustered at the state level and accounts for data autocorrelation.^26,29^ In primary analyses, we analyzed associations between anti-GM policies and mental health symptoms in the entire sample while lagging outcome measurement by 1 survey year to ensure treatment effects were measured after policy enactment. In secondary subgroup analyses, we restricted the sample to GM participants. Statistical significance was defined as a 95% CI excluding 0. Analyses were conducted in R, version 4.3 (R Project for Statistical Computing).

Estimation of treatment effects using difference-in-differences analysis requires assumptions of (1) parallel trends and (2) no anticipation period.^26^ We evaluated the plausibility of the parallel trends assumption (ie, absent anti-GM policies, trends in mental health symptoms would be similar between exposed and comparison states) by reviewing the pretrend coefficients and SEs from an “event study” plot.^26^ Estimates were obtained using a fixed baseline period before the start of the exposure period and calculated as the mean change in mean mental health symptom scores at each time period relative to the baseline period for states exposed to anti-GM policies relative to comparison states. The event study plots averaged the group-time estimates to show treatment effects at different exposure lengths. The “no anticipation” assumption implies that mental health symptoms among participants in states with anti-GM policies would not change before a policy was officially enacted. We expected this assumption to hold as many anti-GM policies were last-minute amendments, often introduced and enacted without prior public knowledge or legislative review.^30^

We performed 3 sensitivity analyses as robustness checks. First, we fit the standard 2-way, fixed-effects difference-in-differences models for comparison purposes. Second, we excluded participants from Idaho, whose policy enactment coincided with the start of the study period. Third, we used an alternative comparison group that included both states that were never exposed to anti-GM policies and states that were not yet exposed by that period.

## Results

The final sample included 8733 participants with 16 576 observations from 44 states and the District of Columbia (median [IQR] age, 32.5 years [26.0-45.0 years]; 2024 cisgender men [23.2%], 2355 cisgender women [27.0%], 2198 gender-diverse adults assigned female at birth [25.2%], 321 gender-diverse adults assigned male at birth [3.7%], 1294 transgender men [14.8%], and 541 transgender women [6.2%]) (Table 1). Individuals reported the following ethnic and racial identities: 311 American Indian or Alaska Native participants (3.6%); 467 Asian participants (5.3%); 413 Black, African American, or African participants (4.7%); 646 Hispanic, Latino, or Spanish participants (7.4%); 133 Middle Eastern or North African participants (1.5%); 25 Native Hawaiian or Other Pacific Islander participants (0.3%); 7884 White participants (90.3%); 179 participants who reported another ethnic and racial identity (2.0%); and 1117 participants who reported multiple ethnic and racial identities (12.8%). Over the study period, 314 participants (3.6%) completed their first questionnaire living in states that enacted anti-GM policies in 2020, 1470 participants (16.8%) in 2021, and 548 participants (6.3%) in 2022; 6401 participants (73.3%) completed their questionnaire in states that did not enact anti-GM policies during the study period. This resulted in 6949 participants (79.6%) in comparison states.

**Table 1.** Characteristics of The PRIDE Study Participants, April 1, 2020, to June 1, 2023

Characteristic	Overall	AQ 2020-enacted, AQ 2021-effective policies	AQ 2021-enacted, AQ 2022-effective policies	Comparison states
Individuals, No.	8733	314	1470	6949
States, No.^a^	45	5	10	30
Age, median (IQR), y	32.5 (26.0-45.0)	32.9 (26.2-46.8)	32.4 (25.6-46.5)	32.5 (26.1-44.5)
Gender identity, No. (%)^b^				
Agender	364 (4.2)	11 (3.5)	49 (3.3)	304 (4.4)
Cisgender man	1464 (16.8)	47 (15.0)	258 (17.6)	1159 (16.7)
Cisgender woman	2342 (26.8)	77 (24.5)	360 (24.5)	1905 (27.4)
Genderqueer	1490 (17.1)	48 (15.3)	229 (15.6)	1213 (17.5)
Man	2237 (25.6)	82 (26.1)	386 (26.3)	1769 (25.5)
Nonbinary	2414 (27.6)	84 (26.8)	393 (26.7)	1937 (27.9)
Questioning	580 (6.6)	18 (5.7)	100 (6.8)	462 (6.6)
Transgender man	1266 (14.5)	51 (16.2)	204 (13.9)	1011 (14.5)
Transgender woman	532 (6.1)	36 (11.5)	107 (7.3)	389 (5.6)
Two-spirit	126 (1.4)	4 (1.3)	27 (1.8)	95 (1.4)
Woman	2417 (27.7)	81 (25.8)	377 (25.6)	1959 (28.2)
Another gender identity	764 (8.7)	29 (9.2)	126 (8.6)	609 (8.8)
Selected multiple gender identities	5004 (57.3)	175 (55.7)	773 (52.6)	4056 (58.4)
Gender groups, No. (%)^c^				
Cisgender man	2024 (23.2)	71 (22.6)	377 (25.6)	1576 (22.7)
Cisgender woman	2355 (27.0)	76 (24.2)	384 (26.1)	1895 (27.3)
Gender-diverse AFAB	2198 (25.2)	69 (22.0)	340 (23.1)	1789 (25.7)
Gender-diverse AMAB	321 (3.7)	10 (3.2)	54 (3.7)	257 (3.7)
Transgender man	1294 (14.8)	53 (16.9)	206 (14.0)	1035 (14.9)
Transgender woman	541 (6.2)	35 (11.1)	109 (7.4)	397 (5.7)
Sexual orientation, No. (%)^b^				
Asexual	1063 (12.2)	41 (13.1)	194 (13.2)	828 (11.9)
Bisexual	2872 (32.9)	106 (33.8)	470 (32.0)	2296 (33.0)
Gay	3031 (34.7)	101 (32.2)	543 (36.9)	2387 (34.4)
Lesbian	2154 (24.7)	72 (22.9)	385 (26.2)	1697 (24.4)
Pansexual	1655 (19.0)	67 (21.3)	266 (18.1)	1322 (19.0)
Queer	4065 (46.5)	124 (39.5)	573 (39.0)	3368 (48.5)
Questioning	332 (3.8)	16 (5.1)	56 (3.8)	260 (3.7)
Same-gender loving	609 (7.0)	18 (5.7)	141 (9.6)	450 (6.5)
Straight or heterosexual	197 (2.3)	8 (2.5)	36 (2.4)	153 (2.2)
Two-spirit	72 (0.8)	2 (0.6)	21 (1.4)	49 (0.7)
Another sexual orientation	466 (5.3)	22 (7.0)	88 (6.0)	356 (5.1)
Selected multiple orientations	4610 (52.8)	158 (50.3)	733 (49.9)	3719 (53.5)
Ethnic and racial identity, No. (%)^b^^,^^d^				
American Indian or Alaska Native	311 (3.6)	16 (5.1)	56 (3.8)	239 (3.4)
Asian	467 (5.3)	7 (2.2)	54 (3.7)	406 (5.8)
Black, African American, or African	413 (4.7)	12 (3.8)	72 (4.9)	329 (4.7)
Hispanic, Latino, or Spanish	646 (7.4)	11 (3.5)	146 (9.9)	489 (7.0)
Middle Eastern or North African	133 (1.5)	4 (1.3)	16 (1.1)	113 (1.6)
Native Hawaiian or Other Pacific Islander	25 (0.3)	1 (0.3)	2 (0.1)	22 (0.3)
White	7884 (90.3)	296 (94.3)	1313 (89.3)	6275 (90.3)
Another ethnic and racial identity	179 (2.0)	6 (1.9)	26 (1.8)	147 (2.1)
Selected multiple ethnic and racial identities	1117 (12.8)	30 (9.6)	181 (12.3)	906 (13.0)
Annual household income, No. (%)				
$0-20 000	1200 (13.7)	46 (14.6)	220 (15.0)	934 (13.4)
$20 001-50 000	2025 (23.2)	97 (30.9)	420 (28.6)	1508 (21.7)
$50 001-100 000	2352 (26.9)	87 (27.7)	407 (27.7)	1858 (26.7)
>$100 000	2639 (30.2)	64 (20.4)	338 (23.0)	2237 (32.2)
Missing	517 (5.9)	20 (6.4)	85 (5.8)	412 (5.9)
Living in a state with Medicaid expansion in 2020, No. (%)	6803 (77.9)	117 (37.3)	589 (40.1)	6097 (87.7)
State-level unemployment rate in 2020, mean (SD), %	7.5 (1.7)	6.8 (1.1)	6.8 (1.3)	7.8 (1.9)

Abbreviations: AFAB, assigned female at birth; AMAB, assigned male at birth; AQ, annual questionnaire; PRIDE, Population Research in Identity and Disparities for Equality.

^a^ “States” refer to all 50 US states and the District of Columbia (Washington, DC). Six states were not represented in our sample due to missing data: Hawaii, Mississippi, Montana, North Dakota, South Dakota, and Wyoming. Because participants could have completed multiple questionnaires during the study period, we used participants’ first completed questionnaire to characterize the sample descriptively.

^b^ Percentages may sum to greater than 100% as participants may have selected more than 1 response category.

^c^ Gender identity groups reflect how participants identified when prompted to select 1 of several mutually exclusive gender identity categories.

^d^ “Another” was a self-report option in which participants could provide write-in responses. Examples for ethnic and racial identity write-in responses included “Jewish,” “Mestizo,” and “multiracial.”

**Figure 1.**
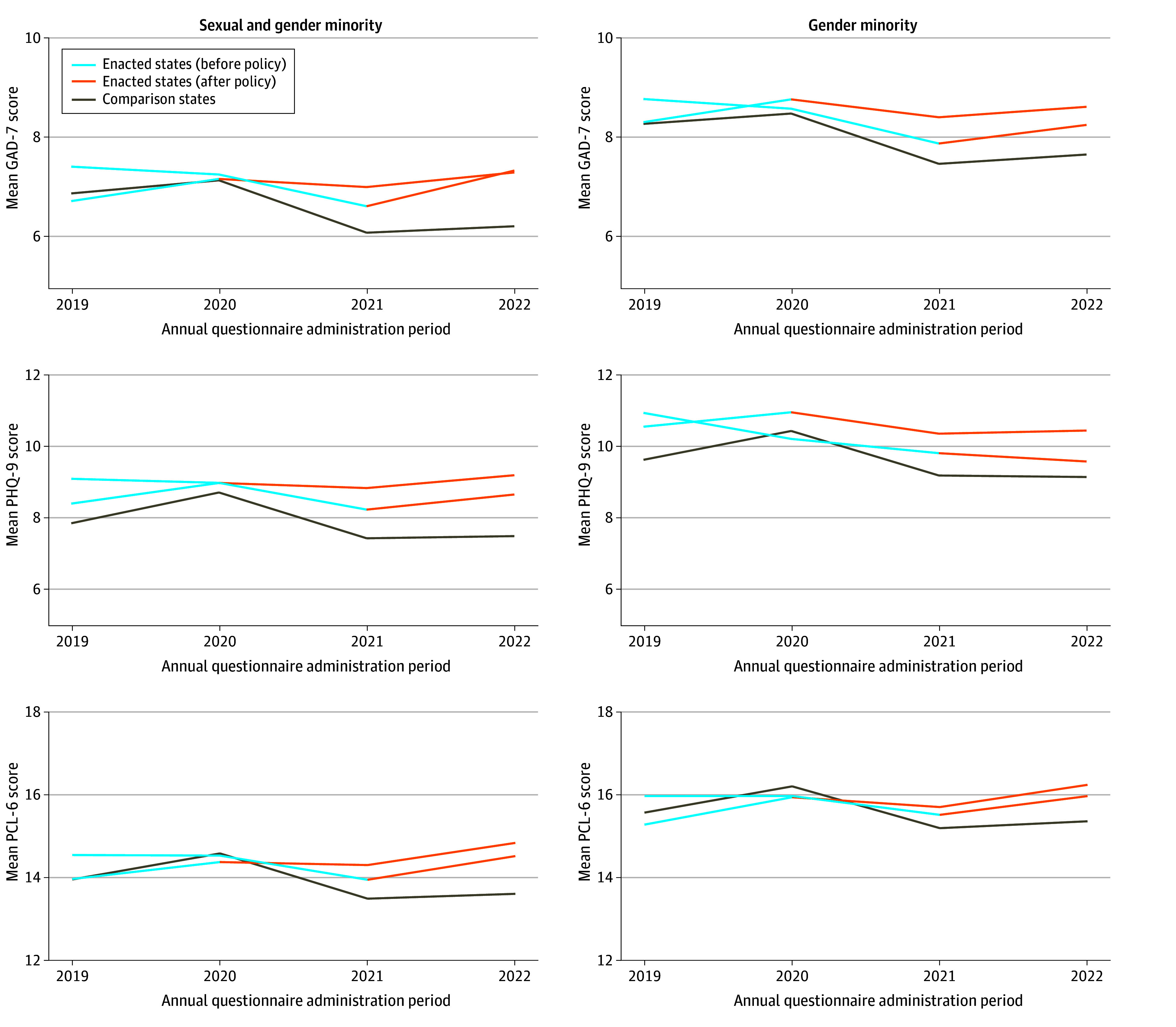
Mental Health Symptoms Among Participants Before and After Enactment of 1 or More State-Level Policies Trends for all sexual and gender minority participants (n = 8733) and gender minority participants (n = 4354) are calculated as an aggregate mean of participants’ individual scores on anxiety, posttraumatic stress disorder, and depression symptom measures for each administration period. Participants who completed annual questionnaires between April 1, 2020, and June 1, 2023, were included; that is, only participants who completed the 2019 annual questionnaire from April 2020 to June 2020 (post-March 2020) were included for that survey year to remove trends related to the onset of the COVID-19 pandemic. GAD-7 indicates 7-item Generalized Anxiety Disorder scale; PCL-6, 6-item Posttraumatic Stress Disorder Checklist; and PHQ-9, 9-item Patient Health Questionnaire.

**Figure 2.**
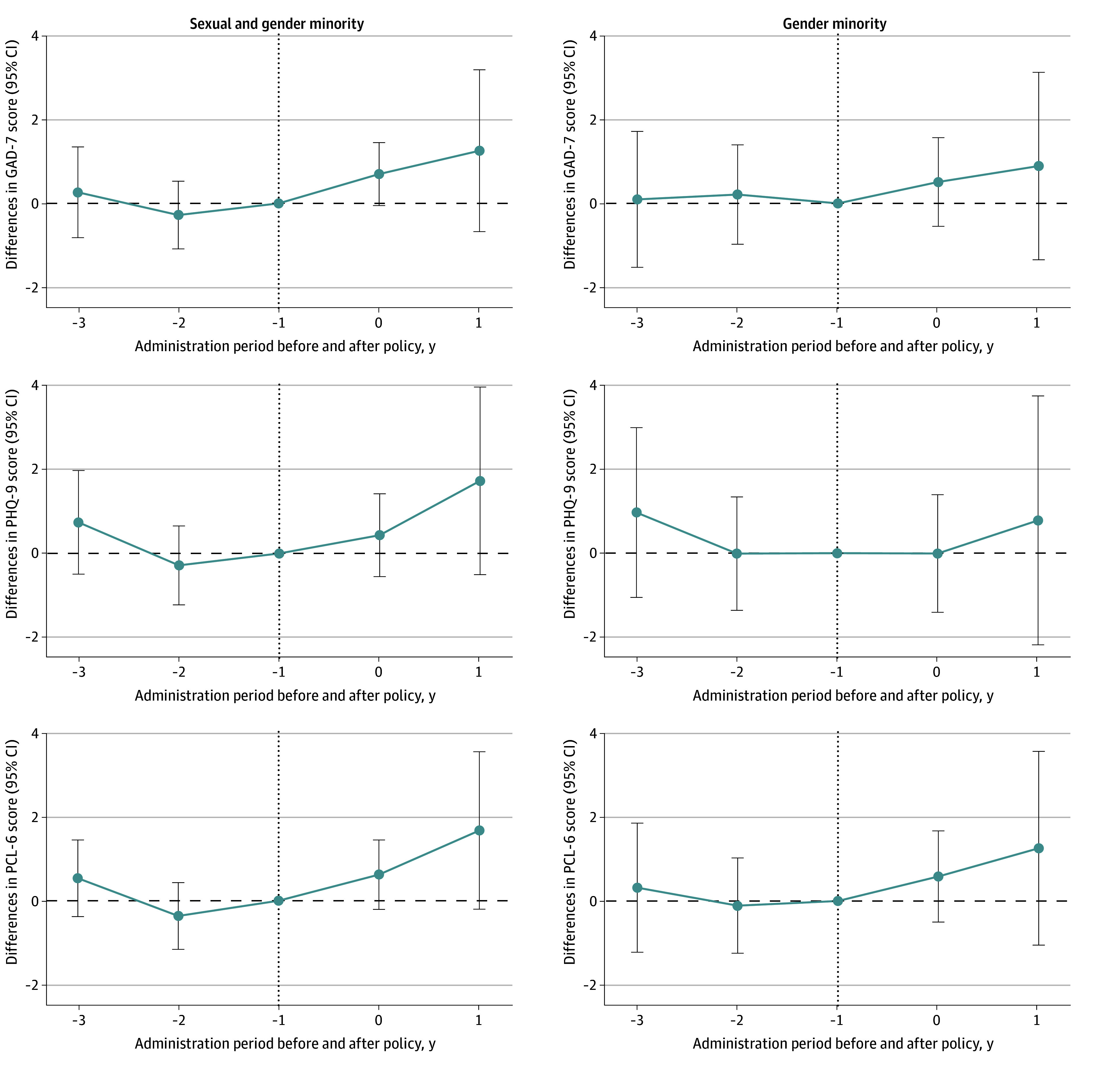
Changes in Anxiety, Depression, and Posttraumatic Stress Disorder Symptoms Over Time Associated With State Policy Enactment Data are drawn from all sexual and gender minority participants and gender minority participants in The PRIDE (Population Research in Identity and Disparities for Equality) Study from April 1, 2020, to June 1, 2023. Dotted lines indicate the period before and after policy enactment. The event study plot was based on aggregated group-time treatment effects averaged at different lengths of the exposure to anti-GM policies. Error bars indicate 95% CIs. GAD-7 indicates 7-item Generalized Anxiety Disorder scale; PCL-6, 6-item Posttraumatic Stress Disorder Checklist; and PHQ-9, 9-item Patient Health Questionnaire.

Figure 1 displays symptoms of anxiety, depression, and PTSD among SGM participants (n = 8733) and GM participants (n = 4354) before and after state-level enactment of 1 or more anti-GM policies. Mean scores are reported across survey year and by policy exposure group among SGM participants in eTable 3 in Supplement 1 and GM participants in eTable 4 in Supplement 1. Gender minority participants scored near or above empirically derived clinical cutoff scores for every mental health measure across study time points. The Figure 2 event study plot indicates that there is plausible evidence that the parallel trends assumption is satisfied in our models.

Table 2 displays difference-in-differences model results. In the overall sample, anti-GM policy enactment was associated with significant increases in anxiety (GAD-7 score, 0.8 points [95% CI, 0.2-1.4 points]) and PTSD (PCL-6 score, 0.8 points [95% CI, 0.1-1.4 points]) symptoms after policy enactment in states with policy enactment compared with states without policy enactment but no significant changes in depression symptoms (PHQ-9 score, 0.6 points [95% CI, −0.1 to 1.4 points]). Among GM adults, anti-GM policy enactment was associated with nonsignificant changes in anxiety (GAD-7 score, 0.6 points [95% CI, −0.2 to 1.4 points]), depression (PHQ-9 score, 0.1 points [95% CI, −0.9 to 1.1 points]), and PTSD (PCL-6 score, 0.7 points [95% CI, −0.2 to 1.6 points]) symptoms after policy enactment in states with policy enactment compared with states without policy enactment.

**Table 2.** Change in Mental Health Symptoms Associated With Anti–Gender Minority State Policy Enactment^a^

Mental health symptom scale	Score change (95% CI)	
	Sexual and gender minority participants (n = 8733)	Gender minority participants (n = 4354)
GAD-7	0.8 (0.2 to 1.4)	0.6 (−0.2 to 1.4)
PHQ-9	0.6 (−0.1 to 1.4)	0.1 (−0.9 to 1.1)
PCL-6	0.8 (0.1 to 1.4)	0.7 (−0.2 to 1.6)

Abbreviations: GAD-7, 7-item Generalized Anxiety Disorder scale; PCL-6, 6-item Posttraumatic Stress Disorder Checklist; PHQ-9, 9-item Patient Health Questionnaire.

^a^ Estimates represent weighted averages in anxiety, depressive, and posttraumatic stress disorder symptoms associated with anti–gender minority state policy enactment in the US for all sexual and gender minority participants and among only gender minority participants in The PRIDE (Population Research in Identity and Disparities for Equality) Study, from April 1, 2020, to June 1, 2023.

Sensitivity analyses (eTable 5 in Supplement 1) using 2-way, fixed-effects models showed smaller associations of anti-GM policy enactment with anxiety and PTSD symptoms in the overall sample. Excluding Idaho and including states that had not yet enacted anti-GM policies in the comparison group yielded consistent results. For the GM subgroup, no significant associations were observed between policy enactment and mental health symptoms.

## Discussion

Our study found that anti-GM policies are associated with increases in anxiety and PTSD symptoms among a large sample of SGM adults across the US. These results are particularly relevant as many states and the federal government increasingly enact policies curtailing GM people’s civil rights. As of writing, over half of US states have enacted GAC bans for young people, and the US Supreme Court will determine the constitutionality of these bans this year.^31^ The current presidential administration has issued executive orders that restrict GM people’s rights.^8^ As anti-GM policies continue to proliferate, the population mental health effects will become increasingly evident.^10,32,33,34,35^

When restricting our analyses to the GM subgroup, we did not observe the same changes in mental health symptoms that we did for all SGM people. This finding contrasts with what we expected: that anti-GM policy enactment would be associated with more pronounced symptom increases among GM people. There are several potential explanations. First, our findings revealed that GM people’s mental health symptoms were high prior to anti-GM policy enactment and across the study period, which is consistent with the broader literature on the substantial burden of mental health challenges among GM people.^36,37,38^ Given the high number of mental health challenges among GM people prior to policy enactment, it is possible that recent enactment of anti-GM policies may not be the best metric for GM people’s disproportionate exposure to stigma and stress.^39,40^ Some anti-GM policies (eg, bathroom bills) had passed or been introduced before The PRIDE Study data collection, and thus, GM people’s mental health may have been affected by these earlier policies.^41^ Alternatively, anti-GM policy enactment could be the actualization, not the catalyst, of GM people’s increased minority stress. That is, anti-GM policies may be enacted because they reflect a preexisting anti-GM state climate—a climate in which GM people are exposed to stigma and disproportionate stress.

Second, the precision of these associations is challenging to estimate without sufficient sample sizes. Our smaller subgroup analyses may not have been large enough to precisely estimate the associations between anti-GM policies and GM people’s mental health symptoms. However, the associations observed in our study were of similar magnitude to the ones estimated in prior research,^42,43,44^ suggesting that small associations between policy and mental health may be highly impactful at the population level^45^ and warrant further investigation.

There are several potential mechanisms through which anti-GM policies may be associated with SGM people’s mental health. First, anti-GM policies may deprive GM people of access to health care and public life (public restrooms, school sports). Researchers, health care professionals, and GM patients have expressed that youth GAC bans have “chilling effects” on adult GAC (ie, health care professionals and patients have been hesitant to engage in such care due to fear of lawsuits, state action, or harassment and violence).^3,46^ Our study’s findings are inconclusive regarding this potential mechanism given that we did not detect significant associations between these policies and GM adults’ mental health symptoms. A second potential mechanism may be that anti-GM policies are associated with worse mental health among SGM people because these policies legally endorse discrimination against SGM people more broadly, creating a hostile anti-SGM state climate. There is evidence for this potential mechanism based on recent survey-based research; SGM adults report feeling less safe in their communities, and 43% report negative health effects from GAC bans, which have largely targeted youths.^15^ In addition, SGM people have reported increases in discrimination and harassment recently amid the increase in anti-GM policies.^47^ Many of the same states that passed anti-GM policies also simultaneously passed policies that directly target SGM people (eg, prohibitions on discussing sexual orientation or gender identity in schools).^2^ Future research examining the potential mechanisms through which these anti-GM policies are associated with SGM adults’ mental health is needed.

### Limitations

This study has some limitations. One is that we examined associations between SGM mental health symptoms and anti-GM policy enactment, not policy implementation. Many anti-GM state policies immediately faced lawsuits and injunctions. Consequently, some anti-GM policies did not go fully into effect or faced legal uncertainty amid judicial proceedings. Although these legal battles may have influenced SGM people’s perceptions and their mental health (eg, could have been perceived as supportive), our lagged approach partly accounts for these associations by ensuring that policy exposure precedes outcome measurement. Furthermore, our analysis relied on the “no anticipation” assumption (ie, that mental health symptoms did not change before policy enactment). This assumption may not hold, specifically for GM adults, and thus may underestimate the results. The data, however, are not suited to test our results’ sensitivity to this assumption given the yearly cadence in which participants are invited to complete The PRIDE Study’s questionnaire. An additional limitation is that SGM people may have moved to more accepting states after policy enactment, changing the composition of state residents.^48^ Future work should examine SGM people’s propensity to move due to anti-GM policies. Another limitation is that our mental health measures were brief and limited. There may have been ceiling effects, particularly for the GM participants who scored high on all measures prior to policy enactment. Finally, time-varying changes such as enactment of other anti-SGM policies during the study period,^49^ the COVID-19 pandemic, and other state policies may have confounded our results.

## Conclusions

In an age of increasing economic inequality, there has been an increase in policies targeting GM people’s health and rights across the country.^50,51,52,53^ Our findings suggest that these anti-GM policies are associated with increased mental health symptoms (particularly anxiety and PTSD symptoms) among SGM adults. As these policies proliferate, it is important to consider how they may affect mental health.

## Notes

Supplement 1. **eTable 1.** Participant Characteristics of Analytic Sample Compared to the Excluded Sample With Missing Mental Health Symptom Data, The PRIDE Study, April 1, 2020 – June 1, 2023 **eTable 2.** List of “Exposed” and “Comparison” States Based on Enactment of Anti-Gender Minority Policies From June 1, 2020 to June 1, 2023 Used in the Primary Difference-in-Differences Model **eTable 3.** GAD-7, PHQ-9, and PCL-6 Scores Across Survey Year and by Policy Exposure Group Among Sexual and Gender Minority Participants in The PRIDE Study, April 2020 – June 2023 **eTable 4.** GAD-7, PHQ-9, and PCL-6 Scores Across Survey Year and by Policy Exposure Group Among Gender Minority Participants in The PRIDE Study, April 2020 – June 2023 **eTable 5.** Results From Sensitivity Analyses Among Sexual and Gender Minority and Gender Minority Participants in The PRIDE Study, April 2020 – June 2023

Supplement 2.
Data Sharing Statement
