# Supplementary material for: US State Policies and Mental Health Symptoms Among Sexual and Gender Minority Adults
Source: JAMA Netw Open. 2025 May 23;8(5):e2512189. doi: 10.1001/jamanetworkopen.2025.12189 (PMC12102706; doi:10.1001/jamanetworkopen.2025.12189)
Supplement: Supplement 1. — eTable 1. Participant Characteristics of Analytic Sample Compared to the Excluded Sample With Missing Mental Health Symptom Data, The PRIDE Study, April 1, 2020 – June 1, 2023 eTable 2. List of “Exposed” and “Comparison” States Based on Enactment of Anti-Gender Minority Policies From June 1, 2020 to June 1, 2023 Used in the Primary Difference-in-Differences Model eTable 3. GAD-7, PHQ-9, and PCL-6 Scores Across Survey Year and by Policy Exposure Group Among Sexual and Gender Minority Participants in The PRIDE Study, April 2020 – June 2023 eTable 4. GAD-7, PHQ-9, and PCL-6 Scores Across Survey Year and by Policy Exposure Group Among Gender Minority Participants in The PRIDE Study, April 2020 – June 2023 eTable 5. Results From Sensitivity Analyses Among Sexual and Gender Minority and Gender Minority Participants in The PRIDE Study, April 2020 – June 2023 [file jamanetwopen-e2512189-s001.pdf]

Supplementary Online Content

Last BS, Tran NK, Lubensky ME, Obedin-Maliver J, Lunn MR, Flentje A. US state policies and mental health symptoms among sexual and gender minority adults. *JAMA Netw Open.* 2025;8(5):e2512189. doi:10.1001/jamanetworkopen.2025.12189

eTable 1. Participant Characteristics of Analytic Sample Compared to the Excluded Sample With Missing Mental Health Symptom Data, The PRIDE Study, April 1, 2020 – June 1, 2023
eTable 2. List of “Exposed” and “Comparison” States Based on Enactment of Anti-Gender Minority Policies From June 1, 2020 to June 1, 2023 Used in the Primary Difference-in-Differences Model
eTable 3. GAD-7, PHQ-9, and PCL-6 Scores Across Survey Year and by Policy Exposure Group Among Sexual and Gender Minority Participants in The PRIDE Study, April 2020 – June 2023
eTable 4. GAD-7, PHQ-9, and PCL-6 Scores Across Survey Year and by Policy Exposure Group Among Gender Minority Participants in The PRIDE Study, April 2020 – June 2023
eTable 5. Results From Sensitivity Analyses Among Sexual and Gender Minority and Gender Minority Participants in The PRIDE Study, April 2020 – June 2023

This supplementary material has been provided by the authors to give readers additional information about their work.

© 2025 Last BS et al. *JAMA Network Open.*

eTable 1. Participant Characteristics of Analytic Sample Compared to the Excluded Sample With Missing Mental Health Symptom Data, The PRIDE Study, April 1, 2020 – June 1, 2023.

**Participant characteristics**	Analytic Sample	Exclude Sample with Missing Outcome
Individuals, No.	8733	2109
States, No.[a]	45	45
Age, median (IQR), years	32.5 (26.0-45.0)	33.3 (26.3-45.6)
Gender Identity, No. (%)[b]		
Agender	364 (4.2)	54 (2.6)
Cisgender man	1464 (16.8)	320 (15.2)
Cisgender woman	2342 (26.8)	540 (25.6)
Genderqueer	1490 (17.1)	318 (15.1)
Man	2237 (25.6)	503 (23.9)
Non-binary	2414 (27.6)	534 (25.3)
Questioning	580 (6.6)	119 (5.6)
Transgender man	1266 (14.5)	296 (14.0)
Transgender woman	532 (6.1)	124 (5.9)
Two-spirit	126 (1.4)	44 (2.1)
Woman	2417 (27.7)	609 (28.9)
Another gender identity	764 (8.7)	146 (6.9)
Selected multiple gender identities	5004 (57.3)	1058 (50.2)
Gender groups, No. (%)[c]		
Cisgender man	2024 (23.2)	481 (22.8)
Cisgender woman	2355 (27.0)	614 (29.1)
Gender-diverse AFAB	2198 (25.2)	499 (23.7)
Gender-diverse AMAB	321 (3.7)	87 (4.1)
Transgender man	1294 (14.8)	298 (14.1)
Transgender woman	541 (6.2)	130 (6.2)
Sexual Orientation, No. (%)[b]		
Asexual	1063 (12.2)	190 (9.0)
Bisexual	2872 (32.9)	652 (30.9)
Gay	3031 (34.7)	727 (34.5)
Lesbian	2154 (24.7)	572 (27.1)
Pansexual	1655 (19.0)	403 (19.1)
Queer	4065 (46.5)	923 (43.8)
Questioning	332 (3.8)	73 (3.5)
Same-gender loving	609 (7.0)	144 (6.8)
Straight/Heterosexual	197 (2.3)	53 (2.5)
Two-spirit	72 (0.8)	27 (1.3)
Another sexual orientation	466 (5.3)	86 (4.1)
Selected multiple orientations	4610 (52.8)	1032 (48.9)
Ethnoracial Identity, No. (%)[b]		
American Indian or Alaska Native	311 (3.6)	94 (4.5)
Asian	467 (5.3)	121 (5.7)
Black, African American or African	413 (4.7)	139 (6.6)
Hispanic, Latino or Spanish	646 (7.4)	205 (9.7)
Middle Eastern or North African	133 (1.5)	31 (1.5)
Native Hawaiian or other Pacific Islander	25 (0.3)	4 (0.2)
White	7884 (90.3)	1827 (86.6)

© 2025 Last BS et al. *JAMA Network Open.*

Another ethnoracial identity	179 (2.0)	47 (2.2)
Selected multiple ethnoracial identities	1117 (12.8)	291 (13.8)
Annual household income, No. (%)		
$0-20,000	1200 (13.7)	149 (7.1)
$20,001-50,000	2025 (23.2)	221 (10.5)
$50,001-100,000	2352 (26.9)	233 (11.0)
$100,000+	2639 (30.2)	292 (13.8)
Missing	517 (5.9)	1214 (57.6)
Living in a state with Medicaid expansion in 2020, No. (%)	6803 (77.9)	1624 (77.0)

AQ, annual questionnaire; IQR, interquartile range; AFAB, assigned female at birth; AMAB, assigned male at birth

[a] "States" refer to all 50 US states and the District of Columbia (Washington, DC). Six states were not represented in our sample due to missing data: Hawaii, Mississippi, Montana, North Dakota, South Dakota, and Wyoming.

[b] Percentages may sum to greater than 100% as participants may select more than one response category.

[c] Gender groups reflect how participants identified when prompted to select one out of several mutually exclusive gender identity categories.

© 2025 Last BS et al. *JAMA Network Open.*

eTable 2. List of "Exposed" and "Comparison" States Based on Enactment of Anti-Gender Minority Policies From June 1, 2020 to June 1, 2023 Used in the Primary Difference-in-Differences Model.

**Enacted during the Annual Questionnaire (AQ) 2020[a] administration period [June 1, 2020 – June 30, 2021]**	**Comparison states**
Alabama, Arkansas, Idaho, Tennessee, West Virginia	Alaska, California, Colorado, Connecticut, Delaware, District of Columbia, Georgia, Illinois, Kansas, Maine, Maryland, Massachusetts, Michigan, Minnesota, Missouri, Nebraska, Nevada, New Hampshire, New Jersey, New Mexico, New York, North Carolina, Ohio, Oregon, Pennsylvania, Rhode Island, Vermont, Virginia, Washington, Wisconsin
**Enacted during the AQ 2021 administration period [July 1, 2021 – May 31, 2022]**	**Comparison states**
Arizona, Florida, Indiana, Iowa, Kentucky, Louisiana, Oklahoma, South Carolina, Texas, Utah	Alaska, California, Colorado, Connecticut, Delaware, District of Columbia, Georgia, Illinois, Kansas, Maine, Maryland, Massachusetts, Michigan, Minnesota, Missouri, Nebraska, Nevada, New Hampshire, New Jersey, New Mexico, New York, North Carolina, Ohio, Oregon, Pennsylvania, Rhode Island, Vermont, Virginia, Washington, Wisconsin

[a] While Idaho enacted their first anti-gender minority policy in March 2020, they were included in the AQ 2020 "treatment" or exposed group for the primary analysis. Sensitivity analyses examined how robust estimates were after excluding participants residing in Idaho from models.

© 2025 Last BS et al. *JAMA Network Open.*

eTable 3. GAD-7, PHQ-9, and PCL-6 Scores Across Survey Year and by Policy Exposure Group Among *Sexual and Gender Minority* Participants in The PRIDE Study, April 2020 – June 2023.

		**AQ 2020-enacted, AQ 2021-effective policies**	**AQ 2021-enacted, AQ 2022-effective policies**	**Comparison states**
Individuals, No.		314	1470	6949
States, No.[a]		5	10	30
Mental Health Symptoms	AQ administration period	Mean, Median, SD	Mean, Median, SD	Mean, Median, SD
GAD-7	2019	6.72, 5, 6.13	7.42, 7, 5.68	6.87, 6, 5.46
	2020	7.17, 7, 5.44	7.26, 6, 5.42	7.14, 6, 5.53
	2021	7.00, 5, 5.89	6.62, 6, 5.37	6.08, 5, 5.26
	2022	7.30, 6, 6.24	7.34, 7, 5.60	6.21, 5, 5.33
PHQ-9	2019	8.43, 8, 5.94	9.13, 8.5, 6.43	7.87, 7, 5.98
	2020	9.01, 8, 6.64	9.01, 8, 6.32	8.74, 8, 6.36
	2021	8.86, 7, 6.78	8.26, 7, 6.33	7.45, 6, 6.03
	2022	9.23, 8.5, 6.91	8.68, 8, 6.30	7.51, 6, 6.07
PCL-6	2019	13.97, 13, 5.46	14.55, 14, 5.23	13.96, 13, 5.20
	2020	14.38, 13, 5.34	14.53, 14, 5.25	14.58, 14, 5.49
	2021	14.30, 13, 5.92	13.95, 13, 5.47	13.49, 13, 5.28
	2022	14.84, 14, 6.44	14.52, 14, 5.52	13.60, 13, 5.37

AQ, annual questionnaire; GAD-7, Generalized Anxiety Disorder scale; PCL-6, Posttraumatic Stress Disorder Checklist; PHQ-9, Patient Health Questionnaire; SD, standard deviation.
[a] "States" refer to all 50 US states and the District of Columbia (Washington, DC).

© 2025 Last BS et al. *JAMA Network Open.*

eTable 4. GAD-7, PHQ-9, and PCL-6 Scores Across Survey Year and by Policy Exposure Group Among *Gender Minority* Participants in The PRIDE Study, April 2020 – June 2023.

		**AQ 2020-enacted, AQ 2021-effective policies**	**AQ 2021-enacted, AQ 2022-effective policies**	**Comparison states**
Individuals, No.		167	709	3478
States, No.[a]		5	10	30
Mental Health Symptoms	AQ administration period	Mean, Median, SD	Mean, Median, SD	Mean, Median, SD
GAD-7	2019	8.31, 7, 7.02	8.78, 8, 5.79	8.28, 7, 5.56
	2020	8.77, 9, 5.46	8.58, 8, 5.64	8.49, 8, 5.59
	2021	8.41, 7, 6.00	7.88, 7, 5.37	7.47, 6, 5.46
	2022	8.62, 7, 6.16	8.26, 7, 5.49	7.66, 7, 5.50
PHQ-9	2019	10.59, 10.5, 5.97	10.98, 10, 6.37	9.66, 9, 6.12
	2020	11.00, 10.5, 6.83	10.25, 10, 6.52	10.47, 10, 6.40
	2021	10.40, 9, 6.67	9.85, 9, 6.45	9.22, 8, 6.24
	2022	10.48, 10, 7.00	9.61, 9, 6.15	9.18, 8, 6.26
PCL-6	2019	15.28, 14, 5.77	15.97, 16, 5.19	15.57, 15, 5.21
	2020	15.94, 15, 5.78	15.97, 16, 5.09	16.21, 16, 5.35
	2021	15.71, 15, 5.78	15.52, 15, 5.33	15.20, 14, 5.30
	2022	16.24, 16, 6.31	15.97, 15, 5.19	15.36, 15, 5.39

AQ, annual questionnaire; GAD-7, Generalized Anxiety Disorder scale; PCL-6, Posttraumatic Stress Disorder Checklist; PHQ-9, Patient Health Questionnaire; SD, standard deviation.
[a] "States" refer to all 50 US states and the District of Columbia (Washington, DC).

eTable 5. Results From Sensitivity Analyses Among Sexual and Gender Minority and Gender Minority Participants in The PRIDE Study, April 2020 – June 2023.

	Mental Health Symptoms	Sexual and Gender Minority Participants	Gender Minority Participants
		Coef (95% CI)	Coef (95% CI)
		n = 8733	n = 4354
**Two-way Fixed Effects**	GAD-7	0.51 (0.21, 0.81)	0.39 (-0.01, 0.78)
	PHQ-9	0.29 (-0.16, 0.74)	0.17 (-0.49, 0.85)
	PCL-6	0.35 (0.05, 0.66)	0.35 (-0.02, 0.72)
		n = 8693	n = 4330
**Excluding Idaho from AQ 2020 exposure group**	GAD-7	0.77 (0.13, 1.40)	0.46 (-0.40, 1.32)
	PHQ-9	0.56 (-0.17, 1.28)	-0.06 (-1.12, 1.00)
	PCL-6	0.76 (0.16, 1.36)	0.58 (-0.25, 1.41)
		n = 8733	n = 4354
**Never- & not-yet-exposed included in comparison group**	GAD-7	0.76 (0.13, 1.39)	0.48 (-0.38, 1.34)
	PHQ-9	0.56 (-0.17, 1.28)	-0.05 (-1.12, 1.03)
	PCL-6	0.73 (0.11, 1.35)	0.56 (-0.28, 1.39)

AQ, annual questionnaire; CI, confidence interval; GAD-7, Generalized Anxiety Disorder scale; PCL-6, Posttraumatic Stress Disorder Checklist; PHQ-9, Patient Health Questionnaire.

© 2025 Last BS et al. *JAMA Network Open.*
