# Supplementary material for: US State Policies and Mental Health Symptoms Among Sexual and Gender Minority Adults
Source: JAMA Netw Open. 2025 May 23;8(5):e2512189. doi: 10.1001/jamanetworkopen.2025.12189 (PMC12102706; doi:10.1001/jamanetworkopen.2025.12189)
Supplement: Supplement 2. — Data Sharing Statement [file jamanetwopen-e2512189-s002.pdf]

Data Sharing Statement

Last. US State Policies and Mental Health Symptoms Among Sexual and Gender Minority Adults. *JAMA Netw Open*. Published May 23, 2025. doi:10.1001/jamanetworkopen.2025.12189

Data
Data available: Yes
Data types: Deidentified participant data, Data dictionary
How to access data: Due to ethical restrictions related to sensitive participant information, study data can be made available on request in accordance with certain data access conditions. Analytic datasets can be made available upon reasonable request by contacting research@pridestudy.org. The data dictionary is already publicly available and can be found here: https://pridestudy.tools/
When available: With publication

Supporting Documents
Document types: None

Additional Information
Who can access the data: Upon reasonable request and in accordance with data access conditions approved by The PRIDE Study team, investigators requesting the data can access the data.
Types of analyses: For specified purposes agreed upon by The PRIDE Study Team.
Mechanisms of data availability: Investigators may need support to access The PRIDE Study data.
